# GLUT1与肺癌及肺癌PET显像的关系

**DOI:** 10.3779/j.issn.1009-3419.2010.02.17

**Published:** 2010-02-20

**Authors:** 向宁 付

**Affiliations:** 430030 武汉，华中科技大学附属同济医院普胸外科 Department of Thoracic Surgery, Tongji Hospital, Huazhong University of Science and Technology, Wuhan 430030, China

葡萄糖转运蛋白(glucose transporter, GLUT)是介导细胞葡萄糖摄取的主要载体, 在人体各组织、细胞中广泛存在。其中GLUT1是已知体内分布最广的转运体, 在各组织中均存在, 在红细胞、血脑屏障、胎盘等中有很高的表达, 与代谢密切相关^[1]^。GLUT1不但参与葡萄糖跨膜转运的正常生理过程, 而且在许多病理情况下出现异常。近年来发现其与恶性肿瘤直接相关, 是目前肿瘤研究的热点之一^[2]^。随着PET技术在肿瘤研究中的应用, GLUT1与肿瘤关系的研究得到了进一步的推动。本文就GLUT1与肺癌及肺癌的PET显像的关系做一综述。

## 1 GLUT1结构、功能和分布

目前研究发现GLUT家族的成员有十几种, 不同的GLUT在体内分布和功能均有差异, 但其结构具有高度的同源性^[3]^。GLUT1为跨膜转运蛋白, 有12个跨膜片断, 整个GLUT1被分为25个节段, 其中13个暴露在细胞内外水溶液环境中的亲水性节段与另外12个埋在细胞膜内的疏水亲脂节段交错排列, 各节段盘绕成螺旋状的筒状结构^[4]^。GLUT1有两种构型, 即外向型和内向型。

GLUT1的主要功能为转运葡萄糖跨过细胞膜进入细胞内, 其外向型亲水性节段有葡萄糖结合位点, 葡萄糖入胞时先与外向型葡萄糖结合位点结合, 然后通过变构再与内向型结合以进行葡萄糖跨膜转运^[5]^。此转运过程是顺浓度差、不消耗能量的易化扩散过程。GLUT1对葡萄糖分子有很高的亲和性, 在调节细胞摄取葡萄糖方面发挥重要作用。

GLUT1主要分布在细胞膜上, 同时在胞浆内也存在富含GLUT1的囊泡, 当细胞对葡萄糖的需求量增多时, GLUT1可从胞内囊泡转移至细胞膜中, 加快细胞对葡萄糖的摄取。GLUT1在细胞中的这种再分布循环运动过程与多种调控机制有关。

## 2 GLUT1的调控

 GLUT1除了在红细胞、脑组织中表达较高, 在肿瘤组织中也常有高表达, 甚至部分炎性组织如炎性肉芽肿中也出现高表达。GLUT1表达的调控受多种因素的影响, 目前研究较多的是乏氧诱导因子-1(hypoxia-inducible factor-1, HIF-1)对GLUT1的调控。

### 2.1 HIF-1对GLUT1表达的调控

乏氧是实体肿瘤发展过程中的普遍现象^[6]^。造成肿瘤乏氧的原因很多, 与实体肿瘤的过快增殖生长引起耗氧量增加、肿瘤组织中血管的生成、血供相对不足等有关^[7]^。HIF-1首先于1992年被Semenza等^[8]^从低氧诱导的细胞核提取物中发现, 是一种转录因子。HIF-1是由HIF-1α亚基和HIF-1β亚基构成的异二聚体^[9]^。乏氧条件下HIF-1α水平升高, 其转录激活区活性增加。乏氧激活的转录因子通过与乏氧反应元件(hypoxiaresponsive recognition element, HRE)结合而达到调控基因的目的。HIF-1α可促进肿瘤组织血管生成和肿瘤细胞葡萄糖代谢相关的蛋白基因的表达。*GLUT1*基因为HIF-1α的靶基因之一, 在肿瘤中表达的上调与HIF-1α的作用相关^[10]^。宋光耀等^[11]^研究GLUT1与HIF-1α的相关性时发现GLUT1 mRNA与HIF-1α mRNA的表达密切相关(*r*=0.6313, *P* < 0.01)。

### 2.2 其它因素对GLUT1的调控

对葡萄糖代谢有调节作用的因素大多也调控GLUT1, 如甲状腺素、雌孕激素、胰岛素、糖原及生长因子等, 均通过不同途径对GLUT1在细胞上的表达及在细胞内的再分布产生影响。如胰岛素可通过有丝分裂原活化激酶(mitogen-activated protein kinase, MAPK)和三磷酸肌醇(phophatidylinositol-3, PI3)激酶信号途径提高GLUT1的表达。有研究^[12, 13]^发现某些抑癌基因能抑制GLUT表达, 减低其活性, 有报道认为p53可下调GLUT1的表达。

## 3 GLUT1与肺癌

无法调控的细胞增殖是肿瘤最主要的特征, 而细胞数的增多导致细胞耗氧量增加, 能量代谢增高。与良性病变及正常的细胞相比, 恶性瘤细胞对葡萄糖的摄取增加, 对葡萄糖的代谢率增加。大量研究^[14-16]^表明恶性肿瘤细胞中往往异常高表达GLUT1。GLUT1与肺癌的关系密切, 在肺癌组织中可检测到GLUT1并且常呈高表达^[17, 18]^。Kurata等^[19]^采用定量RT-PCR技术检测35例肺癌患者的原发性肺肿瘤和正常肺组织, 结果发现GLUT1 mRNA在前者中的表达明显高于后者。由此可见, GLUT1作为介导细胞葡萄糖摄取的主要载体参与了肺癌细胞对葡萄糖的过度摄取过程, 并在肺癌组织的高代谢状态中有着重要作用。

GLUT1在肺癌中的过度表达, 是恶性细胞对其所处的特殊微环境的一种适应性反应。癌组织过快生长、体积迅速增大, 其血供、氧供等能量供应相对不足, 尤癌巢中心部位缺血、缺氧更严重。研究^[20]^表明GLUT1在肺癌中的高表达与HIF-1的正调控作用密切相关。GLUT1的表达与肺癌的大小有关, 肿瘤越大, 其表达越高, 且在癌巢中心的表达比边缘更高^[21]^。GLUT1的表达与肺癌的分化程度也有关^[22]^, 分化程度越低其GLUT1表达越强。此外GLUT1的表达与肺癌的组织学类型有关^[21, 23]^。肺鳞癌中GLUT1染色阳性细胞率常高于腺癌, 小细胞肺癌亦高表达, 但细支气管肺泡癌(bronchioloalveolar lung cancer, BAC)的GLUT1表达低, 可能与BAC较特殊的发生和生长方式导致其肿瘤结构相对松散且生长速度远慢于其它类型的肺癌有关。GLUT1的表达与肺癌的预后相关。王昆等^[24]^对GLUT1表达与Ⅰ期和Ⅱ期非小细胞肺癌预后进行相关性研究发现, 淋巴结转移组的GLUT1的表达明显高于无转移组, 而GLUT1高表达组的生存率较低表达组明显下降, 表明GLUT1的表达与肺癌细胞的侵袭性生长相适应, 肺癌细胞生长越旺盛, 侵袭性越强, GLUT1表达越高, 预后越差。

## 4 GLUT1与肺癌FDG PET显像

正电子发射断层扫描(positron emission tomography, PET)是一种现代核医学影像技术, 利用放射性核素示踪技术显像, 反映脏器的功能、血流和代谢的变化。与CT、MRI不同, PET显像是从分子水平反映肿瘤组织中的生化变化和代谢现象^[25]^。^18^F-脱氧葡萄糖(f luorodeoxyglucose, FDG)是用放射性核素^18^F标记的葡萄糖类似物, 是当前肿瘤PET显像中最常用的显像剂^[26]^。肿瘤细胞浓集FDG, 在PET显像上恶性肿瘤表现为放射性摄取增高的阳性病灶。由于FDG是从肿瘤细胞代谢特性出发设计的显像剂, 从分子水平反映肿瘤的存在和状况, 因此FDG PET显像对于肿瘤具有很高的诊断敏感性。FDG PET最早即被用于肺癌的诊断^[27]^, 其在鉴别肺部肿块良、恶性及评价其恶性程度、判断肺癌淋巴结转移及远处转移、辅助肺癌的临床分期和评估肺癌治疗的疗效及预后等方面均发挥重大作用, 在肺癌中的研究和应用日益广泛。GLUT1与肺癌的FDG PET显像有密切关系, 许多研究^[28, 29]^表明肺癌中GLUT1表达的上调在FDG的摄取率增高中有重要作用。

FDG是葡萄糖的类似物, 具有和葡萄糖相同的进入细胞的途径和方式^[30]^, GLUT1亦介导其跨膜转运。FDG入胞后经己糖激酶作用变成FDG-6-磷酸盐, 因其无法进一步转化为果糖形式而“陷”入肿瘤细胞中, 从而造成FDG的浓集, PET显像学中一般采用半定量分析指标-标准化摄取值(standard uptake value, SUV)来衡量病灶FDG摄取程度。SUV越大, 病灶的FDG摄取量越高。SUV与肿瘤的组织学类型、分化程度、肿瘤的大小及转移等相关。Higashi等^[22]^报道, 在非小细胞肺癌中GLUT1表达阳性的区域与SUV相关(*r*=0.658, *P* < 0.01), GLUT1的高表达加快了肿瘤细胞对葡萄糖和FDG的摄取, 导致SUV高。王昆等^[24]^提出对Ⅰb和Ⅱ期NSCLC患者, 若肿瘤组织中GLUT1高表达且术前FDG PET检查SUV高者, 即使术后证实无淋巴结转移, 也倾向于进行包括化疗在内的综合治疗, 以提高其生存率。

## 5 结语

GLUT1作为介导细胞葡萄糖摄取的主要转运体, 在机体细胞正常的能量摄取与代谢方面具有重要的生理作用, 同时也直接参与了肿瘤等病理过程。GLUT1的表达受多种因素调控, HIF-1有重要的正调控作用, HIF-1是已知的在肿瘤乏氧情况下发挥强大生物活性作用以提高肿瘤组织对缺氧耐受性的重要乏氧诱导因子之一^[31]^。HIF-1α可上调*GLUT1*基因的转录和表达, 从而使其在肿瘤组织中呈高表达。

GLUT1与肺癌关系密切, 在肺癌组织中过度表达, 且与肿瘤的大小、分化程度及组织学类型相关。GLUT1与肺癌的FDG PET显像密切相关, 直接参与了肺癌细胞对FDG的摄取, 其表达与病灶的SUV呈正相关。GLUT1的高表达和高SUV常预示肺癌的恶性程度高、预后差。GLUT1是肺癌组织能量代谢的重要作用因子, 对于进一步揭示肺癌的生物学特性有重要意义
